# Repeatability of Aberrometry-Based Automated Subjective Refraction in Healthy and Keratoconus Subjects

**DOI:** 10.1155/2020/4831298

**Published:** 2020-10-30

**Authors:** Gonzalo Carracedo, Carlos Carpena-Torres, Cristina Pastrana, Ana Privado-Aroco, María Serramito, Laura Batres

**Affiliations:** Department of Optometry and Vision, Faculty of Optics and Optometry, Complutense University of Madrid, Madrid, Spain

## Abstract

**Purpose:**

To compare the intersession repeatability of the Eye Refract, a new instrument to perform aberrometry-based automated subjective refraction, on healthy and keratoconus subjects.

**Materials and Methods:**

A cross-sectional and randomized study was performed. A total of 64 participants were evaluated in the study, selecting one eye per participant randomly. The sample was divided into two different groups: 33 healthy subjects (38.85 ± 13.21 years) and 31 with keratoconus (37.29 ± 11.37 years). Three refractions per participant with the Eye Refract were performed on three different days, without cycloplegia. The repeatability analysis of refractive variables (M, J0, and J45), binocular corrected distance visual acuity (BCDVA), and spent time in refraction was performed in terms of repeatability (*S*_r_), its 95% confidence interval (*r*), and intraclass correlation coefficient (ICC).

**Results:**

There were no statistically significant differences (*P* ≥ 0.05) between sessions in both groups for all refractive variables (M, J0, and J45) and BCDVA. Spent time in refraction was reduced as the sessions went by (*P* < 0.05). The Eye Refract was more repeatable for refractive errors assessment in healthy subjects (M : *S*_*r*_ = 0.27 D; J0 : *S*_*r*_ = 0.09 D; J45 : *S*_*r*_ = 0.06 D) compared to those with keratoconus (M : *S*_*r*_ = 0.65 D; J0 : *S*_*r*_ = 0.29 D; J45 : *S*_*r*_ = 0.24 D), while it was similar for BCDVA.

**Conclusions:**

The Eye Refract offered better repeatability to assess refractive errors in healthy subjects compared to those with keratoconus. Despite measurements being also consistent in keratoconus subjects, they should be treated with caution in clinical practice.

## 1. Introduction

Refraction is probably the most frequent measurement in clinical practice. Subjective refraction is considered the gold standard procedure since it considers both optical and neural aspects of vision [1].

Despite autorefractors allowing us to obtain previous objective refraction as a reference to facilitate subjective refraction, their main limitation is an overestimation of myopia or underestimation of hyperopia [2–9]. Due to the impossibility of using autorefractors to perform accurate and precise refraction as the gold standard, new devices are being developed to perform automated subjective refraction [10–15]. The purpose of these devices is to use an automated algorithm controlled by software to make a subjective adjustment of refraction based on previous objective refraction obtained from autorefraction. Therefore, it is possible to incorporate both objective and subjective refractions into a single instrument.

Currently, the Eye Refract system (Luneau Technology; Chartres, France) is the only commercially available instrument to perform automated subjective refraction [14]. It incorporates a binocular open-field aberrometer that measures objective refraction and a phoropter for subjective adjustment of final prescription. Two of these commercially available instruments in the past are no longer distributed [10, 11, 13].

The accuracy and precision of the Eye Refract to estimate refractive errors has been confirmed in healthy subjects [14, 16]. However, its repeatability to perform automated subjective refraction is still unknown in healthy subjects and other ocular conditions. For this reason, the purpose of the current study was to compare the intersession repeatability of the Eye Refract to perform aberrometry-based automated subjective refraction between healthy and keratoconus subjects. The repeatability analysis was performed for refractive variables, visual acuity, and spent time in refraction.

## 2. Material and Methods

### 2.1. Design of the Study

A cross-sectional and randomized study was conducted in compliance with good clinical practices, institutional review board regulation, and following the tenets of the Declaration of Helsinki [17]. All the participants were voluntarily involved in the study after signing a written consent form, where the purpose and the procedures of the study were explained. The participants were free to leave the study at any time. All the trials were carried out at the University Clinic of Optometry of the Complutense University of Madrid (Spain). The Ethics Committee of the Hospital Clínico San Carlos (Madrid, Spain) approved the performance of the study (code 18/458_R_P).

For each participant, three refractions were performed with the Eye Refract by a single optometrist, without using cycloplegia. The refractions were performed on three different days, during a maximum period of two weeks, depending on the availability of the participants. The intersession repeatability of the Eye Refract was evaluated for refractive variables (M, J0, and J45), binocular corrected distance visual acuity (BCDVA), and spent time in refraction.

### 2.2. Sample

A total of 64 participants were evaluated in the study, selecting one eye per participant randomly (flipping a coin). The sample was divided into two different groups: 33 healthy subjects (38.85 ± 13.21 years) and 31 with keratoconus (37.29 ± 11.37 years). Their demographic characteristics are detailed in Table 1.

The recruitment was carried out to obtain a heterogeneous sample in both groups, whose inclusion and exclusion criteria are detailed in Table 2.

### 2.3. Eye Refract System

**Figure 1:**
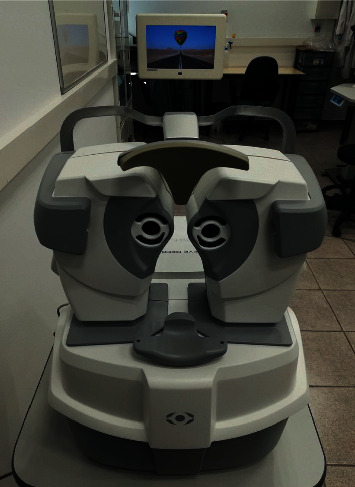
Image of the Eye Refract system and the digital screen used.

The Eye Refract system is a binocular aberrometer combined with a phoropter that performs an aberrometry-based automated subjective refraction (Figure 1). First, the Eye Refract measures the objective refraction from which a subjective adjustment is performed to obtain the final prescription. All these refraction procedures are based on an automated algorithm of the Eye Refract, which is controlled with a digital tablet connected to the system.

Following the manufacturer's instructions, the participants were asked to put their chin and forehead on the supports for this purpose and to look ahead to the fixation image of the digital screen placed at 4 m distance. At this stage, aberrometry was measured in both eyes at the same time with two Hartmann–Shack sensors. These sensors use a near-infrared light of 800 nm, while the pitch of their microlens array of these sensors is 0.1 mm. To calculate the objective refraction, the Eye Refract considers the wavefront metric based on the principle of equivalent quadratic, using the method of paraxial curvature matching proposed by Thibos et al. [18]. This metric considers the Zernike coefficients C^0^_2_ and C^0^_4_ for M determination, C^2^_2_ and C^2^_4_ for J0 determination, and C^−2^_2_ and C^−2^_4_ for J45 determination. The Eye Refract measures the aberrations under physiological pupil size, and it recalculates the values for a pupil size of 3 mm. If the pupil size is inferior to 3 mm, it provides the values for physiological pupil size.

Once objective refraction was measured, the optometrist asked the participants a series of questions provided by the automated algorithm of the Eye Refract to adjust the final prescription. These questions consisted of comparing two spherical or cylindrical lenses, through the “lens 1 or lens 2” method, and the refraction was modified based on their answers until obtaining the final prescription in both eyes.

### 2.4. Analysis of Refractive Variables

The refractive variables were measured in terms of spherical equivalent (M), and both vertical and oblique cylindrical vectors (J0 and J45), based on the method proposed by Thibos et al. [19]. The following formulae were used for the calculations:

  M  = sphere + (cylinder/2)
  J0  = −(cylinder/2) × cos (2 *x* axis)
  J45 = −(cylinder/2) × sin (2 *x* axis)

### 2.5. Measurement of Visual Acuity and Spent Time in Refraction

Since the Eye Refract is a binocular instrument, BCDVA was measured after finishing each refraction. BCDVA was measured through the ocular of the Eye Refract, using the high-contrast (100%) ETDRS chart of the digital screen of the system placed at 4 m distance.

On the other hand, the spent time in refraction was measured with a timer. The time was measured from the start of the objective refraction with the Hartmann–Shack sensors to the final measurement of BCDVA.

### 2.6. Statistical Analysis

The sample size calculations were performed using the Granmo 6.0 software (Institut Municipal d'Investigació Mèdica; Barcelona, Spain), considering the refractive variables as the main ones. An alpha risk of 0.05 and a beta risk of 0.2 in a two-sided test were accepted. Thirty-two eyes were necessary for both groups to recognize as a statistically significant difference greater than or equal to 0.25 D. The common standard deviation was assumed to be 0.50 D.

The statistical analysis was performed using the SPSS Statistics 23 software (IBM; Chicago, Illinois, USA). The intersession repeatability analysis was performed considering the following statistical variables: repeatability (*S_r_*), its 95% confidence interval (*r*), the mean difference between sessions (bias), and its standard deviation (SD). *S_r_* is mathematically defined as the square root of the mean square within-subject standard deviation. *r* is mathematically defined as 2.77 ×  *S_r_*, and it represents the limit value within which 95% of differences between sessions should be [20]. Additionally, the intraclass correlation coefficient (ICC), which represents the degree of agreement between the three repeated measurements, was calculated. According to McGraw and Wong convention [21], ICC analysis was performed using a model of one-way random effects for single measurements.

The normal distribution of all the variables was assessed using the Shapiro–Wilk test. Once the normal distribution of all the variables was confirmed, the one-way analysis of variance (ANOVA) for related samples with Bonferroni correction was performed to check the statistical differences between sessions. A statistical significance of 95% (*P* < 0.05) was established.

## 3. Results

Table 3 summarizes the values of all the variables under study obtained with the Eye Refract in each session and its intersession repeatability results, Table 4 summarizes the differences between sessions and their statistical comparison, and Table 5 summarizes the results of the intraclass correlation analysis.

### 3.1. Refractive Variables

There were no statistically significant differences (*P* ≥ 0.05) between sessions in both groups for all the refractive variables.

Concerning spherical equivalent (M), the Eye Refract was more repeatable in healthy subjects (*S*_r_ = 0.27 D) compared to those with keratoconus (*S*_r_ = 0.65 D), while there was an excellent intersession agreement in both groups (ICC ≥ 0.90).

Similar results were found for cylindrical vectors. The Eye Refract was more repeatable in healthy subjects (J0 : *S*_r_ = 0.09 D; J45 : *S*_r_ = 0.06 D) compared to those with keratoconus (J0 : *S*_r_ = 0.29 D; J45 : *S*_r_ = 0.24 D). Also, both J0 and J45 showed an excellent intersession agreement in both groups (ICC ≥ 0.90).

### 3.2. Visual Acuity

In the case of BCDVA, there were no statistically significant differences (*P* ≥ 0.05) between sessions in both groups, and the Eye Refract showed the same repeatability in both healthy and keratoconus subjects (*S*_*r*_ = 0.04 logMAR). However, the intersession agreement was excellent in keratoconus subjects (ICC = 0.953), while it was decreased in healthy subjects (ICC = 0.748).

### 3.3. Spent Time in Refraction

About spent time in refraction, the one-way ANOVA showed statistically significant differences (*P* < 0.05) between the three sessions in both groups, being the time reduced as the sessions went by. The Eye Refract was more repeatable in healthy subjects (*S*_r_ = 31 s) compared to those with keratoconus (*S*_r_ = 0 : 44 s), while, on the contrary, the intersession agreement was higher in keratoconus subjects (ICC = 0.808) than in healthy ones (ICC = 0.500).

## 4. Discussion

The current study is the first to evaluate the repeatability of the Eye Refract to perform aberrometry-based automated subjective refraction in both healthy and keratoconus subjects. The intersession repeatability analysis showed no statistical differences between the three sessions in both groups for M, J0, J45, and BCDVA, while the values of repeatability for all the refractive variables were better in healthy subjects. Conversely, the values of repeatability for the BCDVA were similar in both groups.

Analyzing the results of spherical equivalent (M), it was found that the Eye Refract was more repeatable in healthy subjects compared to those with keratoconus for all three sessions (see Table 4). Although the *r* value was 2.41 times lower in healthy subjects (*r* = 0.74 D) than in keratoconus subjects (*r* = 1.82 D), the values of ICC were excellent and similar in both groups (see Table 5). This fact would suggest that reporting a single statistical variable could not be enough for repeatability analysis. In agreement with our results, Raasch et al. [22] found that traditional subjective refraction was also more repeatable in healthy subjects (*r* = 1.00 D) than in those with keratoconus (*r* = 10.56 D). They reported higher *r* values than the current study, which could give the impression that the Eye Refract is more repeatable than traditional subjective refraction to assess spherical equivalent. Supporting this idea, Davis et al. [23] also obtained higher *r* values (*r* = 5.70 D) for the sphere with traditional subjective refraction in keratoconus subjects. However, this previous affirmation should be carefully interpreted because there are differences between these two studies and the current one that would not make them comparable, such as sample characteristics, number of sessions, or number of days between sessions.

Concerning other methods of refraction in keratoconus subjects, Shetty et al. [24] showed similar values of ICC for the sphere in both healthy and keratoconus subjects with an adaptive optics visual simulator. Because they only reported values of ICC, we would like to emphasize the importance of analyzing other statistical variables for repeatability analysis. On the other hand, Piñero et al. [25] measured objective refraction with an aberrometer in keratoconus subjects. They found similar values of repeatability for the sphere (*r* = 1.96 *D*; ICC = 0.983) compared to the Eye Refract in the keratoconus group (see Table 3). In healthy subjects, several studies measured objective refraction with different aberrometers, obtaining *r* values between 0.28 D and 0.59 D for the spherical equivalent [16, 26–30]. These values were slightly better than the ones obtained with the Eye Refract in the healthy group (see Table 3).

The intersession repeatability analysis of astigmatism (J0 and J45) showed the same results as the analysis of spherical equivalent. The Eye Refract was more repeatable in healthy subjects compared to those with keratoconus for all three sessions (see Table 4), being the *r* values 3.12 and 3.72 times lower in healthy subjects for both J0 and J45, respectively. The values of ICC were also excellent and similar in both groups (see Table 5). Again, Raasch et al. [22] found that traditional subjective refraction was more repeatable for astigmatism assessment in healthy subjects (J0: *r* = 0.45 *D*; J45: *r* = 0.31 D) compared to those with keratoconus (J0: *r* = 3.86 *D*; J45: *r* = 2.96 D), reaching worse repeatability than the Eye Refract in both groups (see Table 3). Additionally, Davis et al. [23] also obtained higher *r* values for the cylinder (*r* = 2.72 D) with traditional subjective refraction in keratoconus subjects.

With an adaptive optics visual simulator, Shetty et al. [24] showed similar values of ICC for the cylinder between healthy and keratoconus subjects, again with the limitation that they did not analyze other statistical variables for repeatability analysis. With an aberrometer, Piñero et al. [25] found better repeatability in keratoconus subjects for J0 (*r* = 0.45 D) compared to the Eye Refract in the keratoconus group, but worse for J45 (*r* = 1.55 D) (see Table 3). In healthy subjects, several studies measured astigmatism with different aberrometers, obtaining similar or slightly better values of repeatability compared to the current study [16, 26–30].

Although no studies evaluating repeatability of automated subjective refraction in keratoconus subjects were found in the scientific literature, some authors evaluated it in healthy subjects [10–13, 15]. With the first instrument to perform automated subjective refraction, the BV-1000 (Topcon; Tokyo, Japan), Dave and Fukuma [10] found better repeatability in all the refractive variables (M, J0, and J45) than the Eye Refract in the healthy group. Conversely, Sheedy et al. [11] showed similar repeatability with the same instrument compared to the Eye Refract for M and J0 determination, but slightly worse for J45. On the other hand, Perches et al. [12] evaluated a virtual system of automated subjective refraction in only three participants, but by fifty external evaluators per participants. They found better repeatability in the two participants with low astigmatism compared to the one with high astigmatism. Pujol et al. [13] also measured repeatability with a 3D virtual reality instrument of automated subjective refraction, showing slightly better results in terms of *S*_r_ and ICC values for M than the Eye Refract in the healthy group. Finally, Otero et al. [15] used an automated system composed of an open-field autorefractometer and a phoropter with which they obtained better repeatability than the Eye Refract for M and J0 determination, but worse for J45.

In terms of BCDVA, the intersession repeatability analysis showed the same results for both healthy and keratoconus subjects. There were no statistical differences between sessions, and the *r* values were identical (see Tables 3–4). However, the ICC was lower in healthy subjects (ICC = 0.748) compared to those with keratoconus (ICC = 0.953). To our knowledge, only a pair of studies evaluated the intersession repeatability of traditional subjective refraction for high-contrast BCDVA in keratoconus subjects [23, 31]. They found *r* values to be approximately twice compared to the keratoconus group of the current study in addition to a worse ICC (0.769).

One of the advantages of automated subjective refract would be that spent time in refraction is reduced compared to traditional subjective refraction [14, 15]. This is possible due to both objective and subjective refractions that are incorporated into a single instrument. The most remarkable aspect of repeatability analysis was that the spent time with the Eye Refract reduced in both groups as the sessions went by (see Table 3), which could be associated with a learning process by the participants.

The main limitation of the current study was that intersession repeatability of other methods of refraction, in addition to that of the Eye Refract, was not assessed. Therefore, the advantages of the Eye Refract compared to another of these mentioned methods should be carefully treated due to methodological differences between studies.

## 5. Conclusions

The Eye Refract offered better repeatability to assess refractive errors in healthy subjects compared to those with keratoconus. Despite measurements being also consistent in keratoconus subjects, they should be treated with caution in clinical practice.

## Figures and Tables

**Table 1:** Demographic characteristics of the participants in the study.

Group	Number of participants	Age (years)	Age range (years)	Gender (M/F)
Healthy	33	38.85 ± 13.21	[18, 65]	14/19
Keratoconus	31	37.29 ± 11.37	[19, 65]	16/15

**Table 2:** Inclusion and exclusion criteria of the participants in the study.

Inclusion criteria		Exclusion criteria	
Healthy	Keratoconus	Healthy	Keratoconus
Age between 18 and 65 years		Presence of any ocular disease or surgery (apart from keratoconus, in this group)	
Understanding and signing the informed consent		Use of systemic or ocular drugs that could affect the results	
—	With or without intracorneal ring segments	Spherical error higher than ± 15.00 D	
—	Stage I, II, or III, according to Amsler–Krumeich classification	Cylindrical error higher than ± 8.00 D	

**Table 3:** Intersession repeatability of the three sessions in terms of repeatability (*S*_r_) and its 95% confidence interval (*r*) for spherical equivalent (M), cylindrical vectors (J0 and J45), binocular corrected visual acuity (BCDVA), and spent time in refraction.

Variable	Group	Mean ± SD			Repeatability (*S*_r_)	95% confidence interval (*r*)
		Session 1	Session 2	Session 3		
M (D)	Healthy	−1.06 ± 2.54	−1.06 ± 2.48	−1.02 ± 2.50	0.27	0.74
	Keratoconus	−4.76 ± 4.82	−4.45 ± 4.66	−4.42 ± 4.72	0.65	1.82

J0 (D)	Healthy	0.05 ± 0.38	0.06 ± 0.39	0.07 ± 0.35	0.09	0.26
	Keratoconus	−0.71 ± 1.49	−0.78 ± 1.44	−0.79 ± 1.53	0.29	0.81

J45 (D)	Healthy	0.07 ± 0.25	0.06 ± 0.21	0.06 ± 0.24	0.06	0.18
	Keratoconus	−0.05 ± 1.24	0.01 ± 1.29	−0.01 ± 1.28	0.24	0.67

BCDVA (logMAR)	Healthy	−0.21 ± 0.12	−0.22 ± 0.07	−0.23 ± 0.08	0.04	0.12
	Keratoconus	0.04 ± 0.21	0.04 ± 0.20	0.06 ± 0.22	0.04	0.12

Time (min : s)	Healthy	4 : 43 ± 0 : 47	4 : 01 ± 0 : 37	4 : 00 ± 0 : 44	0 : 31	1 : 26
	Keratoconus	5 : 31 ± 1 : 40	5 : 12 ± 1 : 35	5 : 02 ± 1 : 48	0 : 44	2 : 03

**Table 4:** Mean difference between sessions (bias) and its standard deviation (SD) for spherical equivalent (M), cylindrical vectors (J0 and J45), binocular corrected visual acuity (BCDVA), and spent time in refraction.

Variable	Group		Session 1-session 2	Session 1–session 3	Session 2-session 3	ANOVA (*P* value)
M (D)	Healthy	Bias ± SD	0.00 ± 0.07	−0.04 ± 0.08	−0.04 ± 0.05	0.806
		*P* value	>0.999	>0.999	>0.999	
	Keratoconus	Bias ± SD	−0.32 ± 0.18	−0.35 ± 0.17	−0.03 ± 0.13	0.146
		*P* value	0.245	0.166	>0.999	

J0 (D)	Healthy	Bias ± SD	−0.01 ± 0.02	−0.02 ± 0.02	−0.01 ± 0.03	0.607
		*P* value	>0.999	>0.999	>0.999	
	Keratoconus	Bias ± SD	0.07 ± 0.07	0.08 ± 0.08	0.01 ± 0.08	0.511
		*P* value	0.913	0.944	>0.999	

J45 (D)	Healthy	Bias ± SD	0.00 ± 0.02	0.01 ± 0.02	0.01 ± 0.02	0.756
		*P* value	>0.999	>0.999	>0.999	
	Keratoconus	Bias ± SD	−0.06 ± 0.05	−0.05 ± 0.07	0.02 ± 0.06	0.518
		*P* value	0.748	>0.999	>0.999	

BCDVA (logMAR)	Healthy	Bias ± SD	0.01 ± 0.01	0.02 ± 0.01	0.00 ± 0.01	0.425
		*P* value	>0.999	0.621	>0.999	
	Keratoconus	Bias ± SD	0.01 ± 0.01	−0.02 ± 0.01	−0.02 ± 0.01	0.143
		*P* value	>0.999	0.707	0.142	

Time (min : s)	Healthy	Bias ± SD	0 : 22 ± 0.07	0 : 23 ± 0 : 09	0 : 01 ± 0 : 05	0.011^*∗*^
		*P* value	0.007^¥^	0.037^¥^	>0.999	
	Keratoconus	Bias ± SD	0 : 20 ± 0 : 08	0 : 30 ± 0 : 13	0 : 10 ± 0 : 11	0.047^*∗*^
		*P* value	0.080	0.081	>0.999	

The statistical comparison was performed between sessions. ^*∗*^*P* < 0.05, one-way ANOVA for related samples. ^¥^*P* < 0.05, pairwise comparison with Bonferroni correction.

**Table 5:** Intraclass correlation coefficient (ICC) and its 95% confidence interval for spherical equivalent (M), cylindrical vectors (J0 and J45), binocular corrected visual acuity (BCDVA), and spent time in refraction.

Group	Intraclass correlation coefficient (95% confidence interval)				
	M	J0	J45	BCDVA	Time
Healthy	0.989 (0.980, 0.994)	0.935 (0.888, 0.965)	0.929 (0.878, 0.961)	0.748 (0.605, 0.855)	0.500 (0.295, 0.687)
Keratoconus	0.981 (0.966, 0.990)	0.961 (0.931, 0.980)	0.964 (0.936, 0.981)	0.953 (0.917, 0.975)	0.808 (0.686, 0.894)

## Data Availability

The data used to support the findings of this study are available from the corresponding author upon request.
